# Optimization of community-led 3D printing for the production of protective face shields

**DOI:** 10.1186/s41205-020-00089-y

**Published:** 2020-11-23

**Authors:** Peter Chengming Zhang, Yousuf Ahmed, Isra M. Hussein, Edem Afenu, Manon Feasson, Anser Daud

**Affiliations:** 1 Leslie Dan Faculty of Pharmacy, University of Toronto, Toronto, Ontario Canada; 2 Rotman School of Management, University of Toronto, Toronto, Ontario Canada; 3 Faculty of Medicine, University of Toronto, Toronto, Ontario Canada; 4 Institute of Health Policy, Management, and Evaluation, University of Toronto, Toronto, Ontario Canada; 5 Institute of Biomedical Engineering, University of Toronto, Toronto, Ontario Canada; 6 Department of Laboratory Medicine and Pathobiology, Faculty of Medicine, University of Toronto, Toronto, Ontario Canada

## Abstract

**Background:**

As the healthcare system faced an acute shortage of personal protective equipment (PPE) during the COVID-19 pandemic, the use of 3D printing technologies became an innovative method of increasing production capacity to meet this acute need. Due to the emergence of a large number of 3D printed face shield designs and community-led PPE printing initiatives, this case study examines the methods and design best optimized for community printers who may not have the resources or experience to conduct such a thorough analysis.

**Case presentation:**

We present the optimization of the production of 3D printed face shields by community 3D printers, as part of an initiative aimed at producing PPE for healthcare workers. The face shield frames were manufactured using the 3DVerkstan design and were coupled with an acetate sheet to assemble a complete face shield. Rigorous quality assurance and decontamination protocols ensured community-printed PPE was satisfactory for healthcare use.

**Conclusion:**

Additive manufacturing is a promising method of producing adequate face shields for frontline health workers because of its versatility and quick up-start time. The optimization of stacking and sanitization protocols allowed 3D printing to feasibly supplement formal public health responses in the face of a global pandemic.

## Background

With the COVID-19 pandemic causing worldwide personal protective equipment (PPE) supply to diminish, this shortage presented a serious public health concern [1]. PPE includes protective gear such as face shields, masks, gowns, and gloves. Healthcare institutions are at high risk for the transmission of COVID-19, and for clinicians working in direct patient care, PPE is necessary for protection [2]. In Canada, where 115,000 cases have been reported as of July 28, 2020 [3]. Historically, PPE items were generally labelled as single-use. However, during COVID-19, countries such as Canada implemented measures to preserve the supply of PPE. This was a short-term solution to ensure that healthcare professionals remain protected, and included extending the use of PPE items such as masks and face shields [4]. Nevertheless, the ongoing shortage of PPE continued to force frontline workers to improvise, at times even using garbage bags for protection [5].

As manufacturers struggled to keep up with the global demand for PPE [6], the role of 3D printing in augmenting PPE supply increased in popularity and has been widely encouraged by the support of the general community [5, 7, 8]. Institutions such as libraries, universities, and other sources of 3D printers were made idle by the quarantine measures as a response to the pandemic and were readily available to scale up the production of 3D printed PPE. As an integral component of PPE, face shields typically consist of a frame and a transparent plastic sheet attached together, serving as a physical barrier to large droplet transmission. Due to the simplistic, plastic design of face shield frames, 3D printing is a viable option for the production of face shields.

The advantages of 3D printed face shields are worthy of further investigation, particularly those pertaining to automation, consistency between products, and availability of open-source designs and feedback [7–10]. Organizations such as 3Dverkstan, Youimagine, and Prusa have made a variety of 3D PPE designs available [11–13]. In an effort to select the ideal protocol and design to produce face shields, it was imperative that it be cost-effective to print in terms of filament usage, require minimal set-up (i.e., no costly molds or fixtures needed), be optimized for production speed and adequate quality, and be comfortable for healthcare workers to wear for extended periods of time.

Due to the simultaneous development of various 3D printed face shield designs, models were optimized for a different printer models and required adjustment by outside developers during the production process [7]. For this reason, concepts such as stacking, a method used to increase output of face shields in a single printing event, had to be employed to improve efficiency. While this information is readily available to the public, a process of trial and error is necessary to supplement existing literature and serve the practical needs of the initiative.

This report describes the production of 3D printed face shields by a grassroots initiative in Ontario, the province in Canada with the second highest cases of COVID-19 [3], and discusses findings pertaining to the feasibility, sanitization, and stacking processes in manufacturing PPE in the form of 3D printed face shields. By disseminating our findings, we aim to eliminate avoidable costs and challenges for future similar initiatives.

## Case presentation

### Initiative description

Our grassroots initiative served a greater metropolitan area within Ontario, Canada, providing donations of 3D printed face shields to healthcare facilities. Community printers across the province were recruited from institutions and owner-operators and were given standardized Stereolithography (STL) files to print face shield frames. Within the team of printer operators, there was a variety in printing expertise, ranging from academics in engineering to hobbyist owner-operators. When evaluating possible models for our product, the goal was to test and select a design that could be printed by contributors regardless of skill or prior experience. Transparent plastic sheets were obtained from distributors and laser cut to specification. Prior to delivery to end-users, these components were sanitized and packaged in a laboratory setting. Throughout the course of the initiative, 25,000 face shields were delivered to over 165 nonprofit centers and healthcare facilities.

### Design

**Fig. 1:**
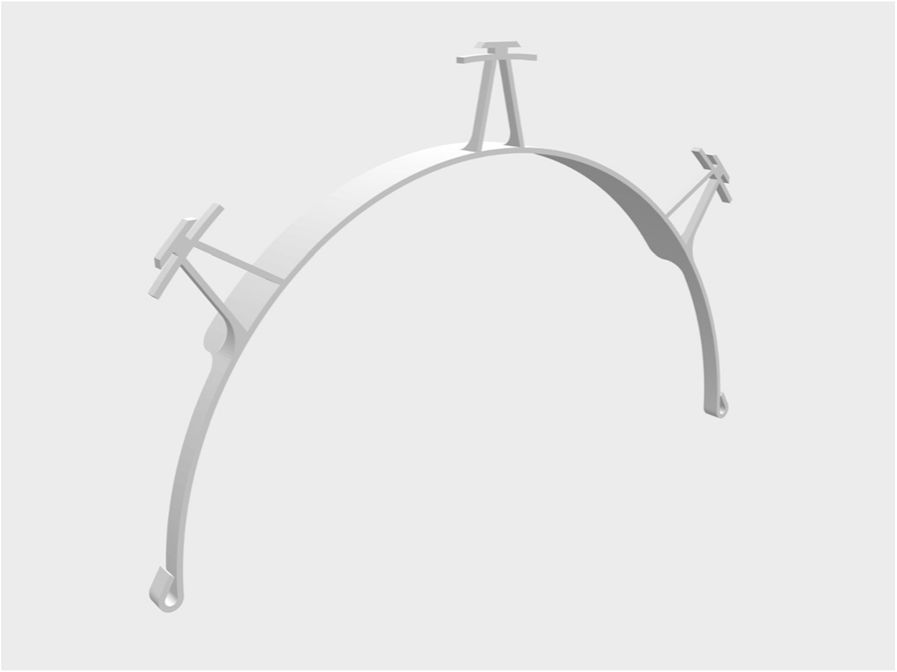
The Shawn Lim face shield design: a computerized image adapted from the Shawn Lim STL file [14] under creative commons license CC-BY-NC-SA

In an effort to rapidly launch this project to meet the PPE demand, the initiative began by donating to a hospital-led PPE drive requesting available 3D printers in the community to print Shawn Lim 3HP v17 face shield frames (Fig. 1) [14]. This design consists of a flat visor frame that wraps around the head with three triangular shaped hooks spaced around the midpoint in front of the frame. These hooks allow for a standard 3-hole punched sheet of plastic to be clipped onto the frame to complete the face shield. Since the frame is designed to rest on the forehead and not the ears, elastics are required to keep the face shield snug to the head. The use of elastics is needed to provide tension and hold the face shield onto the forehead. Moreover, the use of elastics accommodates various head shapes and sizes. The face shield went through multiple design iteration loops until it was optimized for ease of printing and met satisfactory comfort levels for long-term use.

**Fig. 2:**
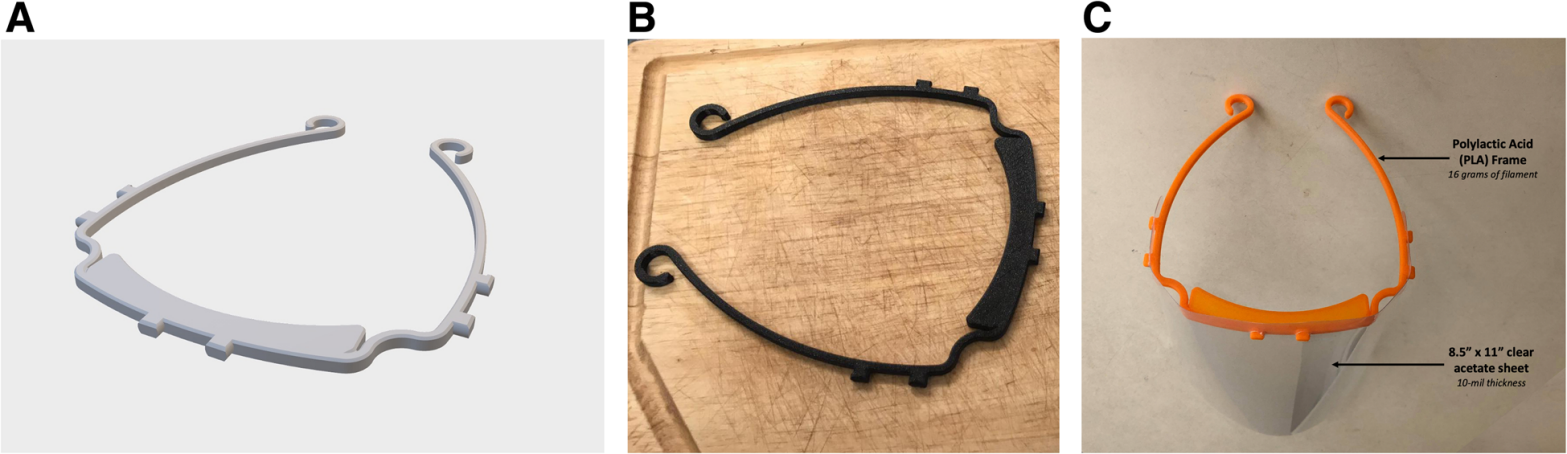
The 3DVerkstan North America face shield design (**a**) computerized image, (**b**) a printed frame and (**c**) a labelled 8.5 X 11 clear acetate shield with an assembled printed frame from 3Dverkstan STL file [15] under creative commons license CC-BY-SA 4.0 International

**Fig. 3:**
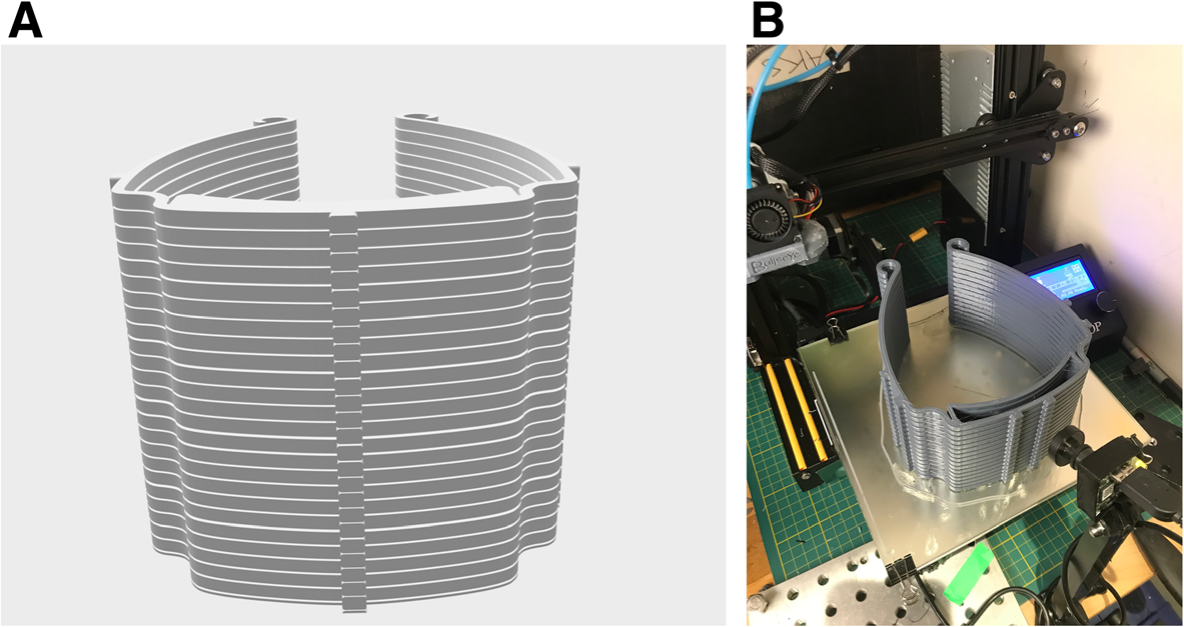
The stacked 3DVerkstan North America face shield design with 0.6 mm spacing developed by our team (**a**) computerized image and (**b**) a printed set, adapted from 3Dverkstan STL file [15] under creative commons license CC-BY-SA 4.0 International

Among newly developed designs, the Swedish 3DVerkstan frame (Fig. 2) was quickly gaining popularity amongst international healthcare institutions [12], as this model was reviewed and recommended by the NIH for use in a clinical setting [16]. The 3DVerkstan design consists of a flat, tapered visor that wraps around the head with 6 rectangular hooks spaced evenly around the front and sides of the frame. These hooks allow for a standard 6-hole punched sheet of plastic to be clipped onto the frame. Unlike the Shawn Lim design which utilizes 3 slim arrow-like hooks placed at the midpoint, the 6 tapered hooks ensure more stability when adhered to a face shield. The 3DVerkstan design shifts some of the tension from the headband to the shield, placing less workload on the elastic. This design is more rigid than the Shawn Lim frame and provided increased stability when attached to a plastic face shield as it utilized 3 additional hooks. The frame is also more conducive to stacking (Fig. 3) compared to other designs due to its broader surface area (3356 mm^2^) and shorter height (5.12 mm), enabling multiple units to be printed on the same printer bed. With these considerations in mind, after supplying the hospital PPE drive, printing production was switched over to the 3DVerkstan design. Print settings for manufacturing 3DVerkstan faceshields are outlined in Table 1.

**Table 1:** 3DVerkstan Print Specifications Ranges [15, 16]

Printer Nozzle Size	0.4 mm	0.6 mm	0.8 mm	1.0 mm	1.2 mm
**Line width**	0.5 mm	0.66 or 0.8 mm	0.8 mm or 1.0 mm	1.0 mm	1.33 mm
**Layer height** **(Layer thickness)**	0.25 mm (standard hotend)	0.3 mm (standard hotend)	0.3 mm (standard hotend) 0.5 mm (high flow hotend)	0.6 mm (high-flow hotend)	0.6 mm (high-flow hotend)
**Wall thickness**	1.6 mm	1.6 mm	1.6 mm	1.6 mm	1.6 mm
**Wall line count**	4	4	4	4	4
**Suggested Print speed settings**	40–50 mm/s	40–50 mm/s	40–60 mm/s	40-70 mm/s	40–125 mm/s

### Stacking

While printing multiple masks simultaneously on the print bed was advantageous, stacking prints was not advised for novice 3D printing operators due to increased difficulty in maintaining consistent print quality. Generally, stacking prints requires more set-up and experimentation with printer-specific settings to mitigate the added risk of stringing, wrapping, or breakage during disentanglement of the stack.

Autodesk Fusion 360 software (Autodesk Inc., Version 2.4.2, California, U.S.) was used to import a version of the open source 3DVerkstan design and develop a 24 stack STL file with a 0.3 mm gap distance. This is because the gap distance between separate face shields for a stack is dependent on factors such as nozzle size and layer height. The gap distance on the STL file should be either equal to or a multiple of the layer height used in slicer settings for printing the stack, in order to prevent stringing and enable easy detachment [17]. Print settings for manufacturing stacked 3DVerkstan faceshields are outlined in Table 2.

**Table 2:** Standardized 3DVerkstan Stacking Settings [15–17]

Printer Nozzle Size	0.4 mm	0.6 mm	0.8 mm	1.0 mm	1.2 mm
**Line/Extrusion width**	0.5 mm	0.66 or 0.8 mm	0.8 mm or 1.0 mm	1.0 mm	1.33 mm
**Layer height**	0.25 (standard hotend)	0.3 mm (standard hotend)	0.3 mm (standard hotend) 0.5 mm (high flow hotend)	0.6 mm (high-flow hotend)	0.6 mm (high-flow hotend)
**Suggested gap spacing**	0.25 mm	0.3 mm	0.3 mm	0.6 mm	0.6 mm
**Wall thickness**	1.6 mm	1.6 mm	1.6 mm	1.6 mm	1.6 mm
**Wall line count**	4	4	4	4	4
**Suggested Print speed settings**	40 mm/s	40 mm/s	45 mm/s	45 mm/s	50 mm/s

NB: It is important to note that the gap distance can be a multiple of the layer height. For example, when attempting to print stacks with a 0.4 mm nozzle using a layer height of 0.25 mm, the gap distance can be 0.25 mm or 0.5 mm

### Procurement of filament and plastic sheets

The design of the face shield involved the use of only two materials: 3D printing filament and transparent plastic sheets. We prioritized minimizing costs, and emphasized ease of production over durability, as the face shields were not intended for prolonged use. We explored standard thermoplastics used for 3D printing that had been discussed by biomaterial engineers and commercial face shield developers in various 3D printing communities. The most cost-effective options for 3D printing filaments were PLA and PETG.

While PLA is marginally cheaper, PETG is more durable and amenable to heat-disinfection. PLA and PETG were observed to have similar tensile strengths of 58 MPa and 56 MPa, respectively [18]. However, PETG has a significantly greater modulus of elasticity and thermal resistance (18 GPa and 80 °C) when compared to PLA (5 GPa and 60 °C) [11].. In addition, PETG is more resistant to various environmental conditions such as sun, rain, and cold, when compared to PLA [19]. Despite these slight mechanical advantages that PETG has over PLA, it was observed that PLA and PETG were both effective options for the production of face shield frames.

The design team experimented with vinyl and acetate sheets for the shield component of the face shield. In addition to cost-effectiveness and ease of production, transparency and stability of the plastic when attached to the frame was a primary consideration. Plastic sheets were cut into 8.5″ by 11″ rectangles and required hole-punch sized cutouts for the frame to sit in. It was found that 10 mm was the minimum sheet thickness that was firm yet pliable enough to adapt to the curved shape of the 3D printed frame. Initially, large vinyl sheets were purchased from plastic manufacturers directly, but these were not well suited for laser cutting due to the production of toxic fumes. Instead, focus shifted to more cost-effective acetate binder covering sheets that were directly sourced from supply store distributors. Ultimately these pre-cut 8.5 × 11 clear acetate sheets of 10 mm thickness were chosen as they only required hole-punching to match the 3D printed frame.

### Disinfection

3D printing is intrinsically a sterile process due to the high temperatures required for production [20]. However, one of the largest challenges posed by 3D printed material designed for use in medical practice, is the re-sterilization and disinfection process required once it has been exposed to the external environment. Such challenges are the result of the relatively low glass transition temperatures of the thermoplastic, eliminating numerous sterilization techniques. The glass transition temperature is the temperature at which the polymer changes from a rigid to a malleable state. This was a significant obstacle to overcome due to the nature of the 3D printed frames being printed by members of the greater community, and the number of hands it passed through during shipping and handling prior to distribution to end-users.

The use of 70% ethanol is effective in inactivating COVID-19 [21, 22]. Alcohol-based antiseptics are regularly used in healthcare settings, are easily accessible, and do not leave behind any residue. This ensures that once the frames are delivered, the facilities using these frames may continue to use their normal disinfectant wipes or alcohol-based products to disinfect after use. For these reasons, we chose to use 70% ethanol as the disinfectant of choice. The developed protocol involved completely submerging the PLA and PETG frames in 70% laboratory-grade ethanol for 10 min to ensure bacteria and viruses were eliminated during disinfection (SARS was seen to be inactivated when in contact with 70% ethanol for 1 min and COVID-19 at 5 min) [23–25]. It is important to note that surface exposure time to ethanol-based products may differ depending on the product, and users should refer to protocol by the United States Environmental Protection Agency when uncertain [22]. Once the frames were placed and covered in a basin containing 70% ethanol, they were left to dry on a disinfected rack lined by diapers. These frames were then placed in a sterile bag to further dry overnight. Throughout this process, frequently changed masks and gloves were worn to avoid contamination. Once completely dry, the disinfected frames were packaged in bundles of 20 into clean resealable plastic bags for distribution.

## Discussion

### Regulatory barriers to face shield production

Given that Health Canada Medical Device Establishment Licence (MDEL) considerations include the processing of a licensing application, as well as in-house production of PPE, we decided to forego licensing in order to meet the direct needs of healthcare workers in a timely manner. Although an MDEL was not obtained, we ensured due diligence in production, decontamination, and communication about the processes involved to ensure that the face shields were reliable and safe for use. In order to do this, the components of the face shield, the 3D printed visor and the plastic sheet, were delivered disassembled to the receiving organization, and a waiver of liability was signed by the organization receiving the donation. Disassembly minimizes further points of contact by volunteers, ensures decontamination is retained, and maximizes the number of face shields that can be transported at a time.

### Quality versus production capacity

Print speed is a key variable associated with optimal face shield quality and production capacity. The 3DVerkstan design’s recommended print settings (e.g. 0% infill, 1.6 mm wall thickness) fostered the printing of face shields at stable speeds (40–60 mm/s), while printing at very high speeds to increase production (60–125 mm/s) resulted in issues such as overheating of face shields frames due to insufficient cooling. In addition, other printing errors such as wrapping, ringing, and weak layer adhesion often occurred [26, 27].

Stacking was a means of maintaining quality while increasing production speed. This method was only carried out by advanced printing operators who had the expertise to troubleshoot and conduct experimental runs until optimal print settings for their printers were identified. It was essential for stacks printed at high speeds to maintain quality without resulting in breaks during detachment.

In future endeavors, we recommend that novice printer operators maximize their printer bed by arranging at least 2 shields on the bed, and printing at comfortable speeds (40–50 mm/s) when using simple printers with small print nozzles (0.4 mm). In addition, to optimize time spent managing printing, we suggest alternate modes of production for novice printer operators, such as printing singles during the daytime and printing stacked face shield frames for overnight prints. Furthermore, the use of post-production treatment protocols, such as sanding or an acetone wash, should be performed to remove 3D printing lines that render the face shield as less visually aesthetic to ensure end-user peace of mind.

### Disinfection troubleshooting

It is important to note that sterilization and disinfection are both decontamination processes, however, they execute different degrees of organismal destruction. While both are essential for proper healthcare delivery, sterilization destroys all microbial life whereas disinfection eliminates many or all pathogenic microorganisms, with the exception of bacterial spores. In this particular case, sterilization is not essential for safe and effective use of the 3D printed face shields, as per CDC guidelines [28].

Unsuccessful techniques that were performed included thermal sterilization (i.e. autoclave and dry heat), laboratory glassware washer, and EtO/H_2_O_2_ gas sterilization. Each technique presented unique challenges. As previously mentioned, PLA and PETG were chosen as the thermoplastic filaments of choice which have a glass transition temperature of approximately 60–65 °C and 80–85 °C [29], respectively. Autoclaving is one of the most rigorous and accessible sterilization techniques, requiring elevated pressure and a sustained temperature of 121 °C for a defined period of time [30]. When the 3D printed frames were exposed to the pre-set autoclave dry plastics cycle conditions, the frames warped and fused together. Furthermore, autoclaving and steam sterilization have been found to decrease the mechanical strength of such plastics [31]. The Laboratory Glassware Washer G 7883, Miele Professional was thereby trialed due to its low temperature settings and sanitization capabilities, however, the results were much the same. Finally, a hot air dryer was trialed at the recommended and verified temperature of 65 °C for 60 min to eliminate potential bacteria and viruses present on the surface. While this method was successful, disinfection was limited to the number of frames that could be disinfected at one time and by the longer exposure time [21]. Alternative sterilization methods, such as low-temperature gas sterilization using EtO or H_2_O_2_, have been validated for use on 3D printed materials [30]. However, these protocols are not cost-effective nor readily available, as their use had been reserved for re-sterilization of N95 masks used by frontline workers.

Bleach, or 10% sodium hypochlorite, is a commonly utilized disinfectant amongst similar PPE initiatives. However, a concern of using bleach is that if the protocol is not followed precisely (e.g. the frames were not thoroughly rinsed with water, the bleach solution is not diluted correctly, or the frames are submerged for an inappropriate amount of time) the solution can cause potential degradation of the material, effectively altering the integrity of the frames [32, 33]. This is in part a consequence of using Fuse Deposition Modeling, which results in a porous structure of the printed material [34, 35]. Bleach is an extremely corrosive agent that degrades even the most resistant materials (e.g. epoxy), must be remade daily, and must be disposed of carefully as it cannot be poured down the sink [33]. In addition, the use of bleach over time has the potential to cause yellowing of the plastic shield, compromising visibility. Furthermore, we discovered that using bleach incorrectly on these items may also cause skin and eye irritations for the user [32]. Based on these findings, it was necessary to find an alternative disinfection protocol that would have less variability.

### Limitations of ethanol

While there are many benefits to using ethanol, there are a few shortcomings that must be taken into consideration. Ethanol is a volatile molecule that evaporates very quickly. Products must be completely submerged for the appropriate amount of time to ensure complete disinfection and should be used in a well-ventilated area. Furthermore, 100% ethanol cannot be obtained by the general public and therefore must be obtained and used in a certified facility. Finally, there are a number of thermoplastics that can degrade from prolonged exposure to various incompatible liquid solvents [34]. For this reason, it is important to select a disinfectant based on the characteristics of the selected plastic.

## Conclusion

The manufacturing and optimization of 3D printed face shields involved several novel considerations. While the role of 3D printing in medicine has become increasingly recognized, this report provides novel insight of its potential capacity in public health through the mobilization of a wider range of community member contributors. The processes of stacking, the cost-benefit optimization in reducing quality in exchange for increased output, and sanitization and disinfection protocols were evaluated and tested in this initiative. It should be recognized that as the COVID-19 pandemic continues, and with future pandemics an inevitability, such community-led efforts may once again become necessary [36, 37]. For this reason, it is important that future grassroots initiatives are well-equipped to provide efficient and effective supplementation of necessary PPE. The importance of such initiatives is also highlighted by the observation that resources that have been prioritized for healthcare communities are regularly unavailable to the public, such as retail or service employees. We hope that the technical lessons learned from this initiative can inform future public health interventions that leverage 3D printing and provide insight for future community-led 3D printing initiatives.

## Data Availability

All data generated or analysed during this study are included in this published article.

## Appendix

### Acronyms

Coronavirus Disease 2019 (COVID-19).

Computer Aided Design (CAD).

Ethylene Oxide (EtO).

Hydrogen Peroxide (H_2_O_2_).

National Institutes of Health (NIH).

Personal Protective Equipment (PPE).

Polyethylene terephthalate glycol (PETG).

Polylactic acid (PLA).

Shawn Lim 3HP V17 design (Shawn Lim).

Stereolithography (STL).

Three-dimensional Printing (3D Printing).

**Print Settings.**

**Shawn Lim 3HP v17 frames.**

Printing a single Shawn Lim frame can take between 15 and 30 min depending on the type of printer, printer settings, and print quality desired. Printers were advised to maximize printer bed capacity by printing 2 frames simultaneously.

Suggested print settings for the Shawn Lim frame [14, 38]:

- 100% infill
- No use of print supports, rafts, or brims
- 0.3 mm layer height (for faster printing)
- 100 mm/sec or higher print speed depending on printer
- Initial layer with a slower speed of approximately 30 mm/s
- Wall thickness 1.6 mm
- Wall line count 4

Advanced print settings were used to guide experienced printers as well as printers who were willing to print stacks overnight. Examples of other advanced print settings that were used by seasoned printers were maximizing printer bed area by printing more face shields on the same bed, printing at maximum speed, and stacking.

**3DVerkstan frames.**

Printing a single 3DVerkstan frame face shield can also take between 15 and 30 min depending on the type of printer, printer settings, and print quality desired. Printers were advised to maximize printer bed capacity by printing 2 frames on the same printer bed.

Suggested print settings for the 3DVerkstan frame:

- 0% infill
- No use of print supports, rafts, or brims
- Initial layer with a slower speed of approximately 20 mm/s
- Wall thickness 1.6 mm
- Wall line count 4

To account for variations in the types of printers used with respect to their specifications, print settings were suggested in ranges.
